# An Interesting Presentation of Pneumomediastinum Secondary to Hyperemesis Gravidarum in the Second Trimester

**DOI:** 10.7759/cureus.48574

**Published:** 2023-11-09

**Authors:** Nida Ansari, Sacide S Ozgur, Robert Giannetti, Faith Powell, Patrick Michael

**Affiliations:** 1 Internal Medicine, St. Joseph's Regional Medical Center, Paterson, USA; 2 Internal Medicine, St. Joseph's University Medical Center, Paterson, USA

**Keywords:** mediastinal emphysema, pregnancy, second trimester, pneumomediastinum, hyperemesis gravidarum

## Abstract

We present a rare case of pneumomediastinum in the setting of hyperemesis gravidarum. Pneumomediastinum is a condition characterized by the presence of air leaking into the mediastinum. Often secondary to trauma, there remains the potential for it to develop spontaneously. This is a time-sensitive diagnosis that requires vigilance for effective treatment. A 21-year-old gravida 1 para 0 female at 15 weeks of gestation with no significant past medical history presented to the emergency department complaining of four weeks of daily nausea and vomiting and two weeks of chest pain, cough, and difficulty breathing. Computed tomography angiography of the chest revealed the presence of subcutaneous air in the mediastinum, and esophageal rupture was ruled out by a gastrografin esophagram. Due to extensive pneumomediastinum and severe metabolic derangements, the patient was admitted to the intensive care unit. A gastrografin esophagram was obtained, which showed no esophageal tear.No surgical intervention was performed, and she was managed with conservative treatment. This case illustrates some of the severe complications of pregnancy. Although pneumomediastinum secondary to hyperemesis gravidarum seen in this patient is rare (the incidence of pregnancy-related pneumomediastinum has been noted to be 1:100,000), it is an important adverse complication that the clinician should keep in mind in pregnant patients with hyperemesis gravidarum. Providing education to patients during the prenatal process can help identify the signs and symptoms of this condition to prevent potentially fatal consequences.

## Introduction

Pneumomediastinum (mediastinal emphysema) is the presence of free air in the mediastinum, mainly originating from the trachea, lungs, esophagus, and peritoneal cavity. Laennec first described pneumomediastinum in 1819 as an outcome of traumatic injury [1]. In 1939, spontaneous pneumomediastinum was reported by Louis Virgil Hamman, after whom the Hamman sign (precordial systolic crepitations and diminution of heart sounds) and Hamman syndrome (spontaneous pneumomediastinum along with subcutaneous emphysema) were named [2].

Pneumomediastinum can occur secondary to chest trauma, surgery, esophageal or tracheobronchial perforation, barotrauma, and mechanical ventilation, or spontaneously due to elevated intra-alveolar pressure precipitated by vigorous exercise, forceful vomiting, coughing, childbirth, use of inhaled recreational drugs, infection of upper airways, asthma, and interstitial lung diseases [3]. Patients with spontaneous pneumomediastinum classically present with chest pain, dyspnea, and subcutaneous emphysema [3]. Pneumomediastinum mostly resolves by itself, with air being reabsorbed gradually unless there is a tension pneumomediastinum, which can be a fatal condition requiring decompression [4].

To date, pneumomediastinum was described during pregnancy secondary to esophageal rupture or spontaneously due to hyperemesis gravidarum because of increased intrathoracic pressure [3,5]. Spontaneous pneumomediastinum in pregnancy is a rare entity with an incidence of less than 1:44.000, and there are very few case reports in the literature [3]. It is imperative to distinguish spontaneous pneumomediastinum from more sinister pathologies, such as an esophageal rupture in pregnancy. Herein, we report a rare case of pneumomediastinum secondary to hyperemesis gravidarum in a 21-year-old female during the second trimester.

This article was previously presented at CHEST Annual Meeting on October 9, 2023.

## Case presentation

A 21-year-old gravida 1 para 0 female at 15 weeks of gestation with no significant past medical history presented to the emergency department (ED) complaining of four weeks of daily nausea and vomiting and two weeks of chest pain, cough, and difficulty breathing. She stated that she was unable to eat due to the vomiting. She endorsed minimal streaks of blood in her sputum. She described the chest pain as constant and moderate in severity, and the shortness of breath was associated with the chest pain. She previously went to the ED for nausea and vomiting two weeks prior to presentation; however, she returned as the symptoms did not resolve.

**Figure 1:**
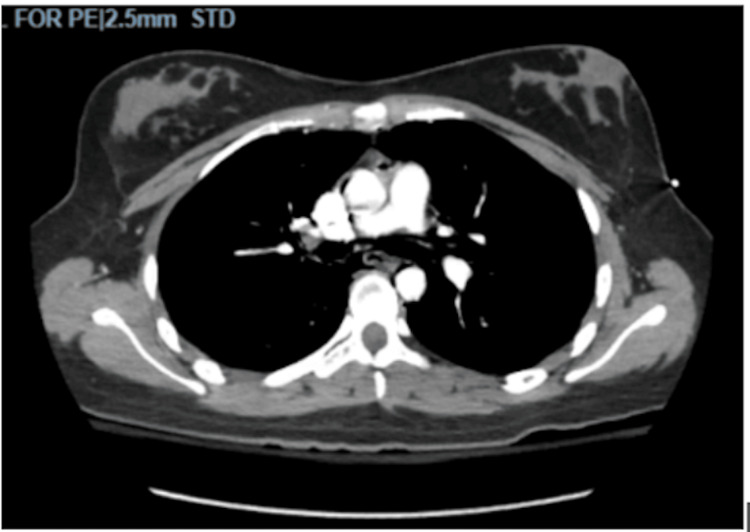
Findings of CT angiography of the chest (sagittal view). There is diffuse subcutaneous emphysema in the neck and pneumomediastinum. There is diffuse esophageal wall thickening, and esophageal perforation cannot be excluded. There is no significant mediastinal or hilar adenopathy. No pulmonary emboli, pulmonary infiltrates, pleural effusion, or pneumothorax can be identified.

**Figure 2:**
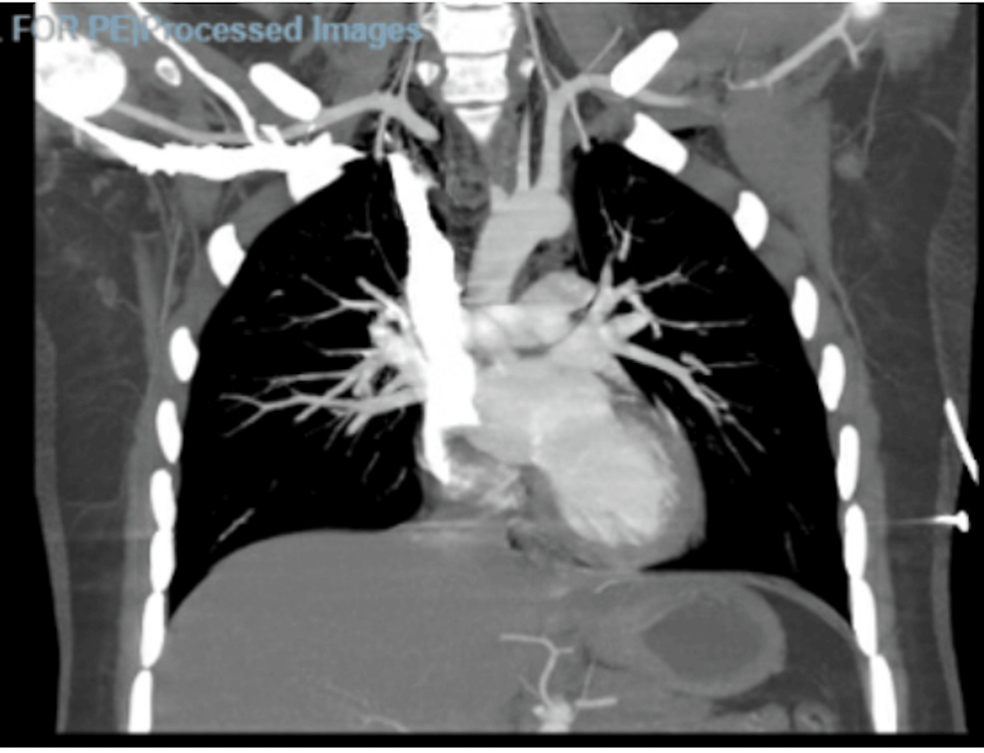
Findings of CT angiography of the chest (coronal view). There is diffuse subcutaneous emphysema in the neck and pneumomediastinum. There is diffuse esophageal wall thickening, and esophageal perforation cannot be excluded. There is no significant mediastinal or hilar adenopathy. No pulmonary emboli, pulmonary infiltrates, pleural effusion, or pneumothorax can be identified.

**Figure 3:**
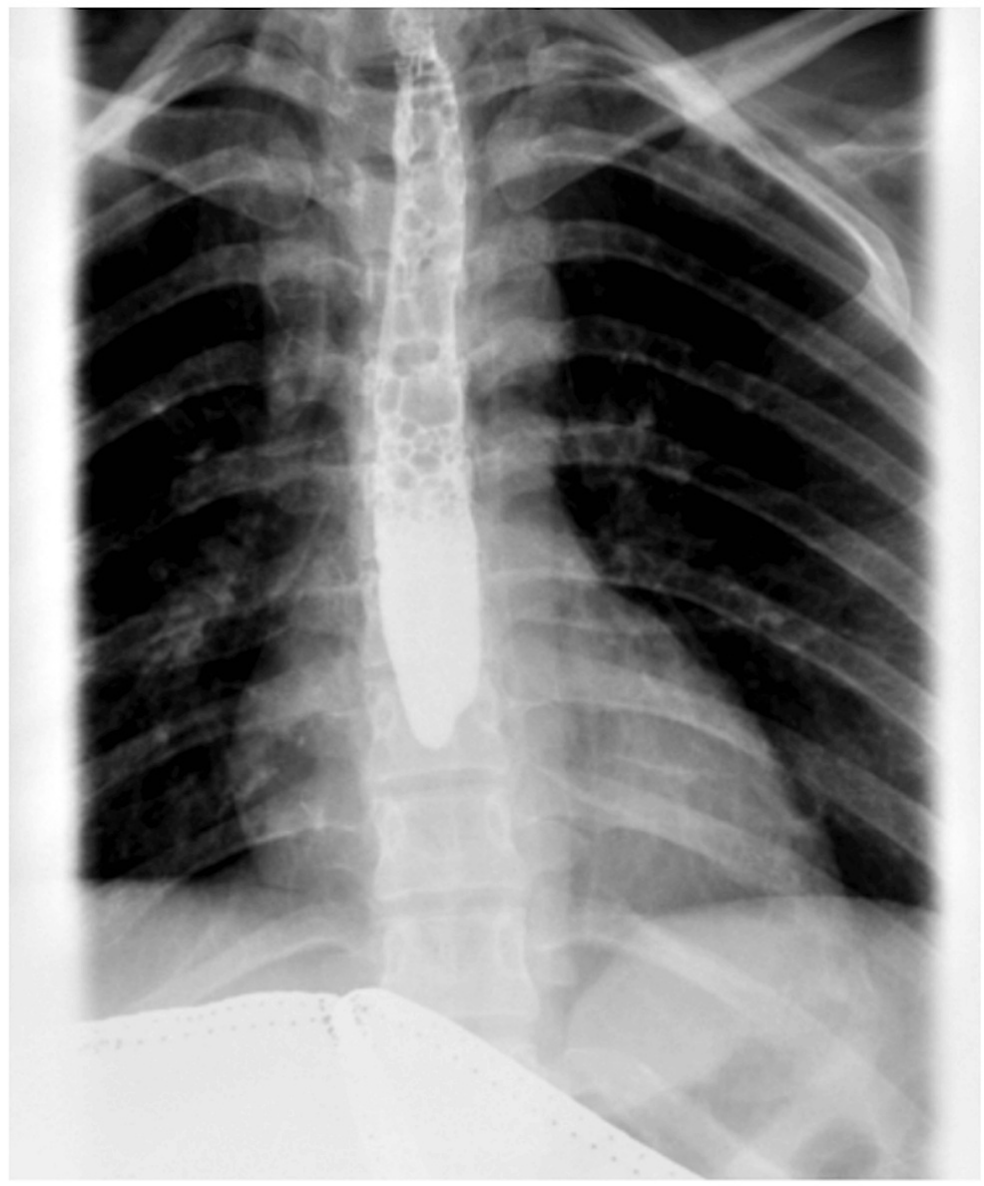
X-ray of the esophagus findings: negative for esophageal tear. Normal in-line flow with peristalsis of the esophagus can be noted.

On physical examination, she was found, the patient was noted to have mild subcutaneous emphysema, and crepitus was noted near the clavicles bilaterally and the neck. She was found to be severely dehydrated and tachycardic in the ED. Labs were significant for several electrolyte derangements, including hypophosphatemia, hyponatremia, and hypophosphatemia. She was also found to have elevated lactate (3.4 mmol/L), leukocytosis (19.2 x 10^3^/mm^3^), transaminitis (AST of 134 unit/L and ALT of 380 unit/L), elevated total bilirubin (4.6 mg/dL), an acute kidney injury (AKI), and elevated D-dimer (1.63 mcg/mL FEU). Given the patient's symptoms, electrocardiography and chest radiography were performed, which showed sinus tachycardia and no radiologic abnormalities, respectively. Computed tomography (CT) angiography was performed due to concern for pulmonary embolism, which showed diffuse subcutaneous emphysema in the neck and pneumomediastinum, diffuse esophageal wall thickening, and negative esophageal tear (Figures 1, 2). Due to a concern for possible esophageal rupture, gastrografin esophagram was obtained, which showed no esophageal tear (Figure 3). Abdominal ultrasound showed the gallbladder to be distended and full of sludge; the gallbladder walls slightly thickened to 2.3 mm with a positive Murphy’s sign. Due to extensive pneumomediastinum and severe metabolic derangements, the patient was admitted to the intensive care unit (ICU).

While in the ICU, Cardiothoracic Surgery, Gastroenterology, and Obstetrics/Gynecology (OB/GYN) were consulted. The patient remained NPO, was started on IV antibiotics, antiemetics, and proton pump inhibitors, and was treated non-surgically. Her electrolytes were replete, and her electrolyte disturbances improved. An obstetrics ultrasound showed a 15-week intrauterine pregnancy with a positive fetal heart rate. While in the ICU, her vomiting improved, and she continued to endorse mild chest pain and nausea. An infectious workup was sent, including blood cultures, urine cultures, complete blood count with differential (CBC), lactic acid, and procalcitonin, and started on antibiotics. The infectious workup did not reveal any infection. Infectious Disease (ID) was consulted, which recommended continuation of the current treatment with antibiotics and fluids. Urinalysis was found to be positive for leukocyte esterase and have a few white blood cells; however, the patient was found to be asymptomatic. However, per ID recommendations, the patient was continued on Zosyn for a possible asymptomatic urinary tract infection. Partial parental nutrition (PPN) was started at a half rate to prevent refeeding syndrome. Nephrology was consulted due to worsening creatinine despite aggressive fluid resuscitation. AKI was believed to be prerenal in etiology secondary to hyperemesis and dehydration. The patient was transferred out of the ICU as her clinic status improved. She was switched from PPN once she was able to tolerate oral intake. Her diet was slowly advanced as she was able to tolerate PO intake. Her electrolyte abnormalities and AKI improved. She was ultimately discharged after six days of inpatient care.

## Discussion

Hyperemesis gravidarum is severe nausea and vomiting experienced during pregnancy. This can often lead to weight loss and volume depletion during pregnancy [6]. While nausea and vomiting are common in pregnancy, hyperemesis gravidarum is present in only approximately 2% of all pregnancies in the United States [6]. A diagnosis that is difficult to treat and is not without its complications [6]. The pathophysiology of hyperemesis gravidarum remains in question; however, some proposed theories are hormone changes, changes in the gastrointestinal system, and genetics [6]. It is proposed that the rise of estrogen contributes to nausea and vomiting. Estradiol has been noted to peak in early pregnancy, often when vomiting is the most present [6]. As levels of estrogen increase, so does the frequency of vomiting [6]. The lower esophageal sphincter relaxes during pregnancy due to estrogen and progesterone, allowing for gastroesophageal reflux disease symptoms, one of which is nausea [6]. Hyperemesis gravidarum has been more notable among women with family members who also experience hyperemesis gravidarum [6].

Typically, patients will have signs of significant dehydration such as kidney area or electrolyte abnormalities [6]. Elevation of hemoglobin or hematocrit can be seen due to hemoconcentration [6]. AKI and electrolyte abnormalities can also be present [6]. Typically, management includes fluid repletion in the setting of dehydration and nonpharmacologic management; however, if refractory, then antiemetics may be utilized.

The most frequent severe complications noted are Wernicke's encephalopathy, electrolyte imbalance, and vitamin K deficiency. In a meta-analysis by Popa et al. [7], only eight articles were obtained when investigating the thoracic complications of hyperemesis gravidarum [7]. Thoracic complications such as pneumothorax, pneumomediastinum, diaphragmatic tears, and thromboembolic events can occur [7]. Pneumomediastinum and pneumothorax are notably more prevalent in younger females younger than 30 years, multiparous, and those with an extensive history of emesis [7]. Spontaneous pneumomediastinum typically presents during the second stage of labor; there have been few reports of pneumomediastinum during pregnancy [7].

Pneumomediastinum is also known as mediastinal emphysema [8]. Pneumomediastinum can be divided into traumatic and spontaneous [8]. The most common etiologies of spontaneous are alveolar rupture and esophageal rupture [8]. Typically, this results from straining against a closed glottis, as seen in a Valsalva maneuver, such as vomiting, thus leading to increased intrathoracic pressure [8]. Findings that may be seen are the Hamman sign, which is auscultation over the pericardium where a crunching or clicking sound would be noted, as well as substernal pleuritic pain, fever, dyspnea, dysphagia, odynophagia, and dysphonia [8]. Lab findings such as leukocytosis can also be seen [8]. Additionally, signs such as the Mackler triad can be seen, including lower thoracic pain, vomiting, and subcutaneous emphysema [9]. This triad was seen in our patient. Typically, subcutaneous emphysema is notably present around the clavicles and neck.

Diagnosis is confirmed by frontal and lateral chest radiography, which would show gas around the pericardium, pulmonary artery, and major aortic branches, as well as continuous diaphragm sign (due to air trapped in pericardium) and extrapleural sign (gas between parietal pleura and diaphragm) [10]. However, CT scan is more sensitive in detecting pneumomediastinum [8,11]. Management is typically conservative and supportive management if it is uncomplicated by esophageal tear, pneumothorax, tension pneumomediastinum, or pneumopericardium [8]. Most cases of pneumomediastinum are self-limiting and resolve within two weeks [9].

The incidence of pregnancy-related pneumomediastinum has been noted to be 1:100,000 [9]. It is rare to find pneumomediastinum concomitantly with hyperemesis gravidarum. Less than 15 case reports could be obtained that report the two phenomena. However, when pneumomediastinum occurs in pregnancy, it typically presents during the second stage of labor [7]. The literature review shows limited reports regarding pneumomediastinum in the first and second trimesters. The increase in intrathoracic pressure secondary to vomiting allows for pneumomediastinum to present.

## Conclusions

Hyperemesis gravidarum is characterized by severe nausea and vomiting in pregnancy, which can cause electrolyte imbalance, weight loss, Wernicke encephalopathy, and pneumomediastinum (free air in the mediastinum) via increased intrathoracic pressure. Spontaneous pneumomediastinum secondary to hyperemesis gravidarum is an extremely rare entity, particularly in the first or second trimester. Distinguishing spontaneous pneumomediastinum from the esophageal rupture via appropriate imaging studies is vital. Monitoring and treating any complications is also crucial in decreasing morbidity and mortality.
